# Enhancing Ecological Functions in Chinese Yellow Earth: Metagenomic Evidence of Microbial and Nitrogen Cycle Reassembly by Organic Amendments

**DOI:** 10.3390/genes17010009

**Published:** 2025-12-22

**Authors:** Han Wu, Juan Li, Jian Long, Hongkai Liao, Kaixiang Zhan, Hongjie Chen, Fenai Lei

**Affiliations:** 1 Guizhou Provincial Key Laboratory for Information System of Mountainous Areas and Protection of Ecological Environment, Guizhou Normal University, Guiyang 550025, China; wuhan_5577@163.com (H.W.);; 2 School of Geography & Environment Science, Guizhou Normal University, Guiyang 550025, China; 3 School of Geography and Resources, Guizhou Normal College, Guiyang 550018, China

**Keywords:** metagenomics, Chinese Yellow Earths, organic amendments, microbial community

## Abstract

Background: Chinese Yellow Earth is a key subtropical agricultural resource in southwestern China; however, its productivity is limited by acidity and poor nutrient retention. This study examined how reduced nitrogen plus organic amendments affect its soil microbial structure and maize yield. Methods: A field experiment with four treatments evaluated reduced nitrogen fertilization amended with rice husk plus rapeseed cake (RS) or RS with biochar (BC). Soil properties (pH, nitrogen, organic matter) and maize yield were analyzed. Metagenomic analysis (NR database) characterized microbial communities, and correlation analysis with Mantel tests identified key relationships. Results: Combined organic amendments under reduced N significantly increased soil pH, nitrogen components, and organic matter, increasing maize yield by 4.41–8.97%. Metagenomics revealed enriched beneficial genera including *Sphingomonas* and *Bradyrhizobium*. Yield positively correlated with nitrate nitrogen and a beneficial microbial cluster containing *Lysobacter* and *Reyranella*, whereas *Steroidobacter* negatively correlated with key fertility indicators. Mantel tests revealed nitrate nitrogen as the primary correlate of functional gene community succession. Conclusions: This study reveals that reduced nitrogen with organic amendments promotes soil improvement and microbial modulation, demonstrating potential as a sustainable practice to maintain crop productivity in Chinese Yellow Earth. The observed trend toward yield improvement underscores its promise and warrants further validation through additional trials. Overall, the findings highlight the beneficial effects of these amendments on soil health and their role in supporting sustainable subtropical agriculture under reduced nitrogen input.

## 1. Introduction

Chinese Yellow Earths constitute a major component of the cultivated land resources in Southwest China, serving as a crucial agricultural resource [1,2]. However, intrinsic constraints, particularly strong acidity and low nutrient retention capacity, render them susceptible to external disturbances [3,4]. Consequently, developing effective management strategies to enhance their fertility has become a priority in agroecological research [3]. Organic amendments are widely recognized as a viable approach to mitigate soil degradation, primarily by increasing soil organic matter content and improving microbial community structure [5].

Nitrogen (N) reduction is a common practice aimed at reducing greenhouse gas emissions [3], improving nitrogen use efficiency (NUE) [6], and lowering production costs. Studies have shown that controlled-release nitrogen fertilizers can minimize nitrogen loss while enhancing wheat yield and NUE [7], Furthermore, reduced N application helps restore the balance of the soil microbial community, increasing beneficial microorganisms and enhancing soil biological activity [8]. Nevertheless, N reduction alone may lead to nutrient imbalance and crop yield reduction. A peanut trial demonstrated that reducing N by 25% and 35% decreased the leaf area index and net photosynthetic rate, potentially causing yield loss, thus highlighting the necessity of prior organic amendment application [9]. This underscores the critical importance of investigating the synergistic effects between N reduction and organic amendments for optimizing soil management strategies.

Under reduced N input, the application of organic amendments such as rice husk and rapeseed cake plays a pivotal role in regulating the soil micro-ecosystem. Research indicates that their combined application significantly alters the soil microbial community structure at both the phylum and genus levels. For instance, rice husk improves soil aeration and promotes the enrichment of phyla like *Chloroflexi*, whereas nutrient-rich rapeseed cake more readily stimulates the growth of copiotrophic groups such as *Proteobacteria* [8]. At the genus level, these practices often significantly increase the relative abundance of functionally important genera like *Sphingomonas* and *Lysobacter*, which are involved in organic matter degradation and biocontrol [10]. The succession of these microbial communities is directly driven by key environmental factors, including soil pH, organic matter, and nitrate nitrogen content [11]. Correlation analyses further reveal significant positive correlations between the relative abundance of specific beneficial bacterial genera and crop yield, thereby directly linking microbial dynamics to agricultural output [12]. More importantly, the input of these organic amendments profoundly regulates the abundance of key functional genes involved in the soil nitrogen cycle. For example, it can enhance the abundance of the nitrogen-fixing gene *nifH* and the nitrification gene *amoA*, while also influencing the ratio between the denitrification genes *nirK* and *nosZ*, thereby modulating nitrogen use efficiency and loss potential [13]. Mantel tests indicate that changes in these functional genes are closely associated with the aforementioned environmental factors. Collectively, these relationships help elucidate a conceptual cascade originating from the amendments: “Environmental Factor Modulation → Microbial Community and Functional Reshaping → Crop Yield Impact” [14]. Deciphering this cascade is essential for optimizing combined organic and inorganic fertilization strategies and achieving sustainable agricultural development.

Building on this foundation, the present study investigates typical yellow soils in Southwest China by integrating metagenomic sequencing with microbial ecological network analysis. It is designed to address two pivotal scientific questions: (1) How do the synergistic effects of N reduction combined with specific organic amendments (rice husk and rapeseed cake, with or without biochar) quantitatively alter key soil physicochemical properties, including pH, organic matter content, and nutrient availability? (2) Can a multi-layered analytical framework—correlating physicochemical indicators with crop yield, linking microbial genera abundance to these indicators, and applying Mantel tests to connect functional gene profiles with environmental variables—identify the principal drivers and elucidate their integrated effects on crop yield? The findings are expected to establish a theoretical foundation and offer technical support for advancing precise, efficient, and sustainable soil management in Chinese Yellow Earth regions.

## 2. Materials and Methods

### 2.1. Study Area Overview

The field experiment was conducted at a key agricultural demonstration area located in Diba Village, Yangliutang Town, Shibing County, within the Qiandongnan Miao and Dong Autonomous Prefecture of Guizhou Province, China (108°12′59″ E, 29°59′49″ N). The site is situated on sloping farmland at an elevation of 677.5 m above sea level, within the transitional ecotone between the Yunnan Guizhou Plateau and the eastern hilly plains, characterized by typical low-hill terrain of central Guizhou. The region experiences a humid subtropical monsoon climate with distinct seasons. Based on 30-year meteorological averages, the area has an mean annual temperature of 15.5 °C, receives approximately 1600 mm of annual precipitation, has a frost-free period of 280 days, a total annual sunshine duration of 2647.6 h, and a total solar radiation of 127.4 kcal·cm^−2^.

### 2.2. Plant-Based Organic Amendments and Soil

The experimental soil was classified as Chinese Yellow Earth. Its initial physicochemical properties were as follows: pH 4.62, total nitrogen (TN) content of 0.80 g/kg, total phosphorus (TP) content of 0.57 g/kg, available phosphorus (AP) of 25.47 mg/kg, soil organic matter (SOM) of 22.34 g/kg, ammonium nitrogen (NH_4_^+^-N) of 1.72 mg/kg, and nitrate nitrogen (NO_3_^−^-N) of 14.32 mg/kg.

The maize (*Zea mays* L.) cultivar used was Xinzhongyu 801 (Qianshenyu 2011011), procured from the local Soil and Fertilizer Station. This cultivar demonstrates vigorous seedling emergence, characterized by purple leaf sheaths and green leaf margins. All agricultural waste materials (rapeseed cake and rice husk) were locally sourced from farmers within the study area, while the biochar was provided by the same station. The electrical conductivity, pH, total carbon, total nitrogen, and total phosphorus contents of these organic amendments (rice husk, rapeseed cake, and biochar) are detailed in Table 1.

### 2.3. Experimental Design

A field trial was conducted from April to September 2024 following a randomized complete block design with four treatments: (1) CK (Control), receiving local conventional fertilization (N: 302.36 kg/ha, P_2_O_5_: 104.75 kg/ha, K_2_O: 78.7 kg/ha); (2) DP, with no organic amendments but a reduced nitrogen rate (40% at seedling stage and 20% at flare-opening stage compared to CK); (3) RS, applying rice husk (8000 kg/ha) and rapeseed cake (2250 kg/ha) on the DP reduced-nitrogen basis; and (4) BC, applying rice husk (8000 kg/ha), rapeseed cake (2250 kg/ha), and biochar (2011 kg/ha) also on the DP reduced-nitrogen basis. Each treatment had three replicates, resulting in 12 experimental plots. Individual plots measured 5 m × 7.2 m (36 m^2^) and were separated by 1 m-wide buffer rows. Maize was planted at a density of 150 plants per plot (6 rows × 25 plants/row) with 20 cm intra-row and 120 cm inter-row spacing (east-west orientation). Prior to sowing, organic amendments were incorporated into the soil by excavating 20 cm-deep trenches, evenly distributing the materials, backfilling, and thorough mixing. Standard weed control and conventional agronomic practices were maintained throughout the growing season.

Fertilization was applied three times according to local practices: a basal application on 25 April 2024 (with all amendments incorporated once), followed by seedling fertilizer (hole-placement on 27 May 2024) and flare-opening fertilizer (similarly applied on 1 July 2024). Nitrogen and phosphorus were supplied as urea (46% N) and superphosphate (12% P_2_O_5_), respectively. Detailed rates are provided in Table 2.

Rhizosphere soil samples at maturity were collected using a five-point sampling method per plot, with soils from the five points blended into one biological replicate. Due to practical analytical constraints, three biological replicates per treatment (i.e., three independent plots) were selected for metagenomic sequencing. Consequently, a total of 12 metagenomic libraries (3 per treatment) were sequenced. All samples were processed independently during DNA extraction and sequencing. Background soil samples were used solely for basic chemical property analysis and were not sequenced.

### 2.4. Sample Collection and Determination

#### 2.4.1. Sample Collection

Soil samples (0–20 cm depth) were collected using a five-point sampling method both before fertilization (for background assessment) and at crop maturity. For fresh soil analysis, approximately 150 g of homogenized soil was sieved in the field (2 mm mesh) to remove root fragments, stored in airtight bags within ice-cooled containers, and subsequently preserved at −4 °C for quantification of inorganic nitrogen species (NO_3_^−^-N and NH_4_^+^-N). A parallel subsample was air-dried at ambient temperature, then ground and sieved to specific particle sizes for standard physicochemical analysis. An additional subsample designated for microbial community analysis was immediately flash-frozen on dry ice and transported under cryogenic conditions for metagenomic sequencing.

#### 2.4.2. Determination Method

Soil pH was determined using a Lichen pH-100B meter at a soil-to-water ratio of 1:2.5 (*w*/*v*). Soil NH_4_-^+^N and NO_3_^−^-N contents were quantified by indophenol blue spectrophotometry and ultraviolet spectrophotometry, respectively, following extraction with 2 mol·L^−1^ KCl. Air-dried, ground, and sieved samples were analyzed for: soil organic matter (via K_2_Cr_2_O_7_-H_2_SO_4_ oxidation and ultraviolet spectrophotometry), total phosphorus (by HClO_4_-H_2_SO_4_ digestion and the molybdenum blue method), and available phosphorus (using HCl-NH_4_F extraction). For the organic amendments, total carbon and total nitrogen were determined with an Elementar vario MACRO cube analyzer (Elementar, Germany), while total phosphorus was measured after H_2_SO_4_-H_2_O_2_ digestion via the molybdenum blue method. NH_4_^+^-N was analyzed using a standard soil agro-chemical analysis method [15], while all oter paeameters were determined according to established analytical procedures for soil agriculture [16].

Corn yield was determined by a whole-plot harvest method. The key procedures included: selecting and measuring the area of a representative plot, harvesting all ears and recording fresh weight, shelling to obtain fresh grain weight and moisture content, converting to dry weight, adjusting to a standard moisture content of 14% (wet basis), and finally calculating yield per unit area (e.g., kg/ha).

### 2.5. Metagenomic DNA Extraction and Sequencing

Genomic DNA was extracted from soil samples using the QIAGEN DNeasy PowerSoil Pro Kit according to the manufacturer’s protocol. DNA integrity and purity were assessed by 1% agarose gel electrophoresis and a NanoDrop One spectrophotometer, respectively, with absorbance ratios (A_260_/A_280_ of 1.8–2.0 and A_260_/A_230_ ≥ 2.0) and concentration (Qubit 4.0 fluorometer) meeting the following quality thresholds: concentration ≥ 10 ng/μL, total mass ≥ 1 μg, and no significant contamination.

Libraries were prepared using the ALFA-SEQ DNA Library Prep Kit through optimized fragmentation (300–500 bp), end repair and A-tailing, adapter ligation, purification, and PCR amplification. The final libraries were evaluated on a Qsep400 system for fragment size distribution (peak: 350–550 bp) and quantified (Qubit 4.0) to ensure a concentration ≥ 15 nM. Qualified libraries were subjected to paired-end 150 bp (PE150) sequencing on an Illumina platform at Guangdong Magigene Biotechnology Co., Ltd. (Guangzhou, China).

Libraries were prepared using the ALFA-SEQ DNA Library Prep Kit through a series of steps: optimized fragmentation to 300–500 bp, end repair/A-tailing, adapter ligation, purification, and PCR amplification. The final libraries underwent quality assessment on a Qsep400 system for fragment size distribution (peak: 350–550 bp) and were quantified using a Qubit 4.0 fluorometer to ensure a concentration ≥ 15 nM. All libraries meeting these criteria were then subjected to paired-end 150 bp (PE150) sequencing on an Illumina platform at Guangdong Magigene Biotechnology Co., Ltd. (Guangzhou, China).

Open reading frames (ORFs) were predicted from the assembled scaftigs using Prodigal (v2.6.3; -*p* meta), retaining ORFs with a minimum length of 90 bp. Quality-filtered reads were then aligned to the predicted gene catalog using BBMap for abundance quantification, with read counts normalized by gene length. Differential gene abundance analysis was performed on the normalized data using DESeq2 (v1.38.3). Specific comparisons were made between each treatment (DP, BC, RS) and the control (CK) to identify fertilization-responsive genes, applying significance thresholds of FDR < 0.05 and |log_2_FoldChange| > 1. For taxonomic annotation, gene sequences were aligned against the NCBI NR database using DIAMOND (e-value ≤ 1 × 10^−10^) and classified using the Lowest Common Ancestor (LCA) algorithm in MEGAN. Additionally, to identify genes involved in the nitrogen cycle, sequences were specifically aligned against the KEGG database (v2023.2) using DIAMOND.

### 2.6. Statistical Analysis

In all analyses involving multiple hypothesis testing (e.g., α-diversity comparisons, PERMANOVA, and correlation analyses), *p*-values were adjusted using the Benjamini Hochberg method to control the false discovery rate. All statistical analyses were conducted in R(version 4.5.0). Initial hypothesis testing utilized the stats package, employing independent-samples *t*-tests for pairwise comparisons and one-way analysis of variance (ANOVA) followed by Duncan’s post hoc test for multi-group comparisons. Prior to conducting ANOVA, the assumptions of normality (ShapiroWilk test) and homogeneity of variances (Levene’s test) were verified for key variables (e.g., soil pH, total nitrogen, yield). Data meeting these assumptions (all *p* > 0.05) were analyzed parametrically; otherwise, the non-parametric Kruskal Wallis test was applied. To mitigate the influence of sequencing depth, species and functional gene abundances were normalized to relative abundances. Microbial community analyses, including α-diversity calculation and non-metric multidimensional scaling (NMDS) based on Bray Curtis dissimilarities, were performed using the vegan package. Visualizations, such as bar plots of dominant bacterial phyla and heatmaps of yield-associated functional genes, were generated with ggplot2. Correlation analyses encompassed Spearman’s rank correlation (using the Hmisc and psych packages) and Mantel tests (vegan package). These results were integrated and visualized in a comprehensive heatmap using the linkET package. All analyses were performed in R version 4.5.0 with the following package versions: ggplot2 v3.4.4, Hmisc v5.1.1, psych v2.3.9, vegan v2.6.4, and linkET v0.0.7.

## 3. Results

### 3.1. Soil Nutrient Content and Crop Yield

**Figure 1:**
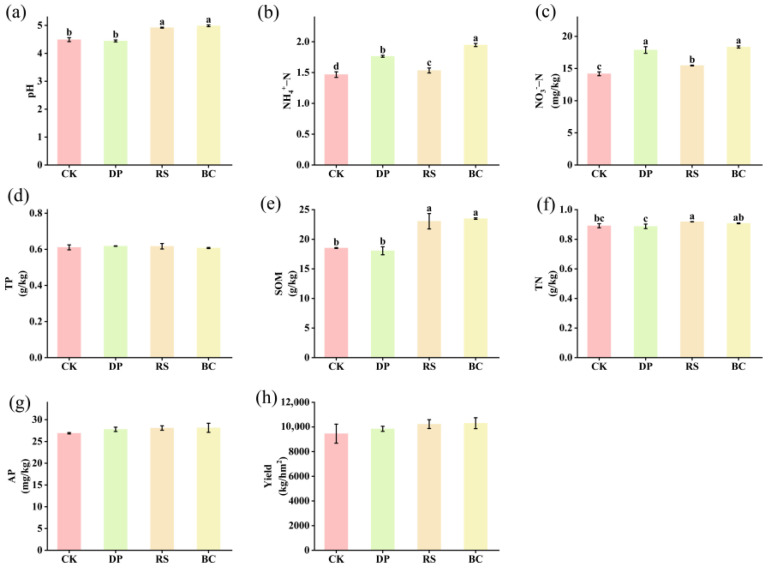
Soil properties and maize yield under different treatments: (**a**) pH, (**b**) ammonium nitrogen (NH_4_^+^-N, mg/kg), (**c**) nitrate nitrogen (NO_3_^−^-N, mg/kg), (**d**) total phosphorus (TP, g/kg), (**e**) soil organic matter (SOM, g/kg), (**f**) total nitrogen (TN, g/kg), (**g**) available phosphorus (AP, mg/kg), and (**h**) yield (kg/km^2^). Note: Different lowercase letters above bars denote statistically significant differences among treatments (*p* < 0.05, one-way ANOVA). Bars lacking letters indicate no significant difference.

The application of organic amendments significantly improved pH, NH4^+^-N, NO3^−^-N, TN, and SOM contents in maize rhizosphere soil (Figure 1). Compared to the control (CK), BC and RS treatments significantly increased soil pH by 11.13% and 9.58%, respectively (*p* < 0.05). Treatments BC, RS, and DP significantly increased soil NH_4_^+^-N and NO_3_^−^-N contents by 9.10–32.74% and 9.10–29.41%, respectively (*p* < 0.05). Furthermore, BC and RS treatments significantly increased soil TN content by 1.84% and 3.04%, respectively (*p* < 0.05). Additionally, BC and RS treatments significantly increased soil SOM content by 26.20% and 26.85%, respectively. The corn yield across treatments was as follows: CK, 9456.48 ± 44.28 kg/ha; DP, 9847.22 ± 118.27 kg/ha; RS, 10,227.77 ± 205.56 kg/ha; and BC, 10,304.62 ± 255.31 kg/ha. These results demonstrate that organic amendment treatments (particularly BC and RS) increased yield while significantly enhancing soil pH, nitrogen content, and organic matter content.

### 3.2. Changes in Soil Microbial Community Diversity

**Figure 2:**
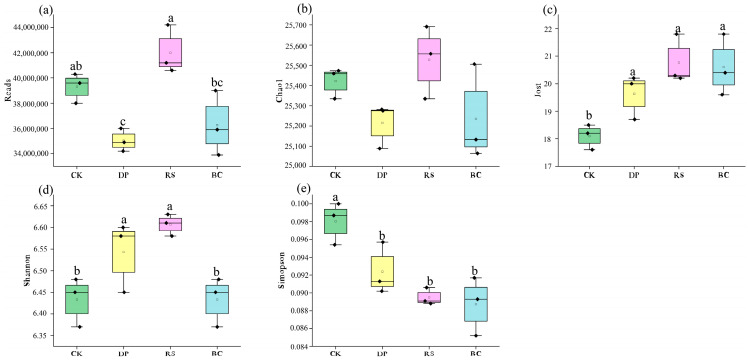
Comparison of Alpha Diversity Indices of Microbial Communities among Different Groups. (**a**) Number of Reads, indicating the sequencing depth of each sample; (**b**) Chao1 index, estimating the total species richness in the community; (**c**) Jost index, effective number of species, based on Hill numbers; (**d**) Shannon index, comprehensively reflecting species richness and evenness of the community; (**e**) Simpson index, reflecting community dominance, which is more sensitive to common species. Bars lacking letters indicate no significant difference.

Metagenomic sequencing of all soil samples was successful, revealing a species richness of 25,064 to 25,691 species per sample. A summary of α-diversity indices is provided in Figure 2. The application of organic amendments significantly influenced the α-diversity of the soil microbial community (*p* < 0.05). Specifically, the Jost index increased significantly under the BC (13.81%) and RS (14.76%) treatments compared to CK. In contrast, the Simpson index was significantly reduced under the BC (9.51%), RS (8.69%), and DP (5.74%) treatments. The Shannon index was significantly higher in the BC (3.26%) and RS (2.70%) treatments. No significant differences were detected for the Chao1 index among treatments. The significant reduction in the Simpson index suggests that organic amendments enhanced community evenness.

**Figure 3:**
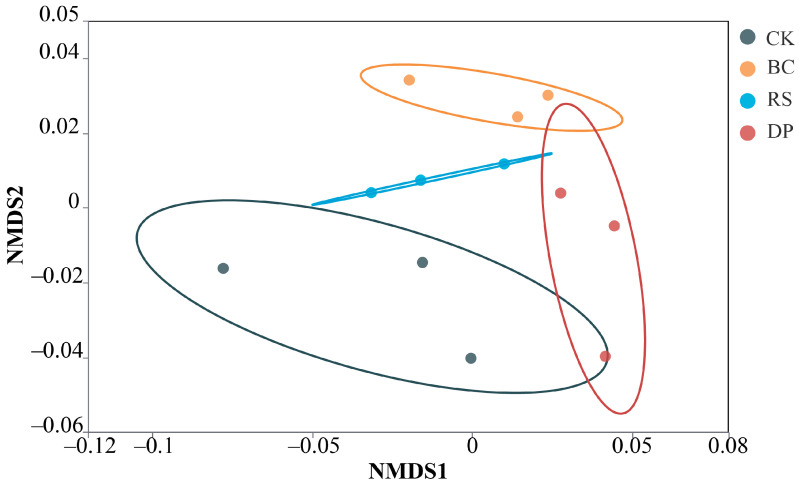
NMDS of community composition based on relative abundance across experimental treatments.

To investigate the effects of different fertilization treatments on rhizosphere microbial community structure, non-metric multidimensional scaling (NMDS) was performed based on Bray Curtis dissimilarity after total sum scaling (TSS) normalization. After 200 iterations, the two-dimensional ordination yielded a stress value of 0.11, indicating a reliable representation of the data, with NMDS1 values ranging from −0.12 to 0.08 and NMDS2 values from −0.06 to 0.05. Treatments were denoted as follows: CK (control, gray), DP (red), RS (blue), and BC (orange). The NMDS ordination revealed distinct spatial separation among treatments (Figure 3), and PERMANOVA confirmed significant differences in community structure across treatment groups (*p* = 0.011).

### 3.3. Organic Amendments Alter Maize Rhizosphere Microbial Community Structure

Based on a systematic investigation of the effects of organic amendments on the maize rhizosphere microbial community structure, this study elucidates the microbiological pathways through which organic amendments promote crop growth from the perspective of community structure. Analysis of the microbial community structure revealed that organic amendments significantly altered the composition and distribution of rhizosphere microorganisms. Taxonomic annotation of maize rhizosphere soil microorganisms against the NCBI NR database identified a total of 4 kingdoms, 36 phyla, 185 classes, 383 orders, 829 families, 3538 genera, and 25,691 species. Domain-level analysis indicated that bacteria predominated across all treatments, with relative abundances of 65.85% (CK), 65.88% (DP), 65.81% (RS), and 65.44% (BC). The relative abundances of Archaea and Eukarya ranged from 0.52% to 0.64% and 0.016% to 0.019%, respectively, with the remainder comprising unclassified taxa.

**Figure 4:**
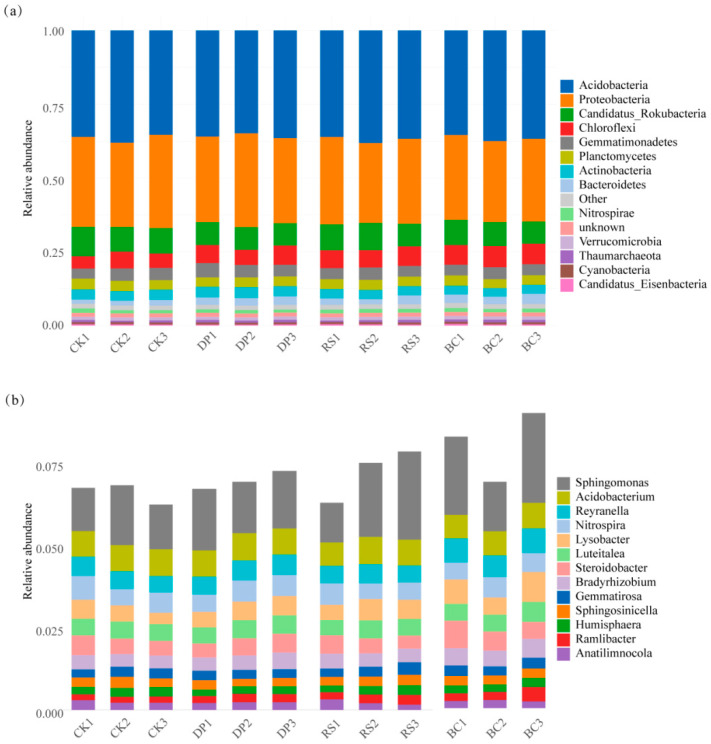
Taxonomic composition profiling of the dominant microbial taxa. (**a**) Relative abundance of the top 15 bacterial phyla; (**b**) Genus-level ranking of the 15 most abundant taxa. Values represent mean abundance (%).

At the phylum and genus levels, different fertilization treatments induced specific successional changes in the microbial community. Analysis of the top 15 dominant phyla and genera (Figure 4a,b) revealed that *Acidobacteria* was the most abundant phylum: 24.08% (CK), 23.67% (DP), 24.41% (RS), and 24.01% (BC). Compared to CK, the BC treatment significantly increased the abundance of *Proteobacteria* (*p* < 0.05), while the RS treatment significantly increased the abundance of *Gemmatimonadetes* (*p* < 0.05). To accurately visualize the distribution of known taxa, “other/unknown” groups potentially containing unclassified species or sequencing artifacts were excluded from the analysis. At the genus level, *Sphingomonas* exhibited the highest relative abundance (CK: 1.49%; BC: 2.20%; RS: 2.04%; DP: 1.72%), followed by *Acidobacterium* (CK: 0.80%; BC: 0.75%; RS: 0.78%; DP: 0.81%). The BC treatment significantly enhanced (*p* < 0.05) the relative abundances of *Reyranella*, *Lysobacter*, *Steroidobacter*, and *Ramlibacter*. These structural changes observed at different taxonomic levels provide a basis for understanding the ecological functions of the microbial community.

### 3.4. Effects of Organic Amendments on the Abundance of Nitrogen-Cycling Functional Genes

**Figure 5:**
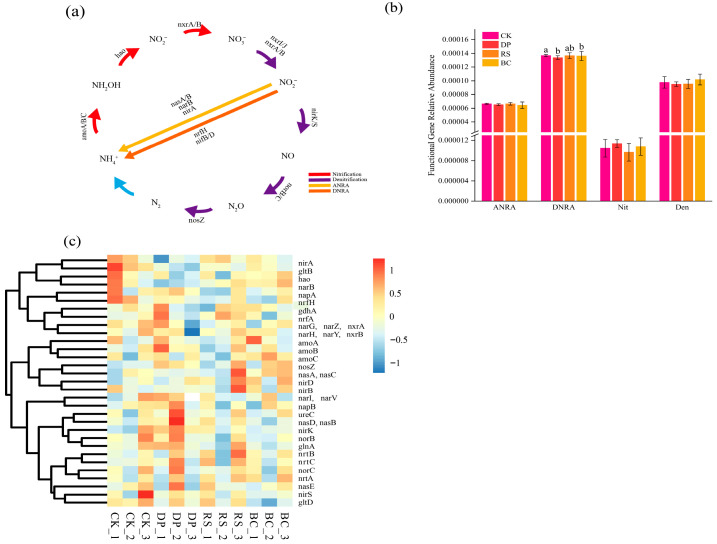
Nitrogen cycling processes, functional gene abundances, and cluster analysis. (**a**) Schematic diagram of key nitrogen cycling processes. (**b**) One-way ANOVA of the relative abundances of five nitrogen cycling functional genes across different treatments. Bars lacking letters indicate no significant difference. (**c**) Cluster analysis of the nitrogen cycling functional genes.

Figure 5a illustrates four microbe-driven nitrogen transformation pathways in soil: nitrification oxidizes ammonium to nitrate (Nit); assimilatory nitrate reduction to ammonium converts nitrate into ammonium for biosynthesis (ANRA); dissimilatory nitrate reduction to ammonium also reduces nitrate to ammonium (DNRA); and denitrification progressively reduces nitrate to gaseous nitrogen compounds (Den). These processes collectively regulate the transformation and fate of nitrogen in soil, with ANRA and DNRA pathways promoting nitrogen retention, while the Den process leads to nitrogen loss. As shown in Figure 5b, analysis of the relative abundance of nitrogen cycle functional genes revealed that different fertilization treatments only significantly affected the relative abundance of DNRA (*p* < 0.05), while no significant effects were observed for other nitrogen cycle functional genes (ANRA, Nit, Den). The detailed grouping, KEGG gene constituents, and ecological functions of these genes are summarized in Table S1. Specifically, the CK treatment showed the highest relative abundance of DNRA genes (mean: 0.01404%), which was significantly higher than that in DP (0.01327%), BC (0.01344%), and RS (0.01363%) treatments.

Through cluster analysis of the relative abundance of nitrogen cycle genes, six distinct functional modules were identified (Figure 5c): Cluster 1 (ammonia oxidation denitrification complete pathway module) (containing 7 genes, including *amoA*, *amoB*, *amoC*, *nosZ*, *nasA*, *nasC*) integrates ammonia oxidation and terminal denitrification functions, representing the complete pathway from ammonia oxidation to denitrification; Cluster 2 (hydroxylamine oxidationassimilatory reduction association module) (containing 4 genes, including *hao*, *narB*, *nirA*, *gltB*) connects hydroxylamine oxidation with assimilatory reduction processes; Cluster 3 (nitrate nitrite conversion core module) (containing 8 genes, including *narG*, *narZ*, *nxrA*, *narH*, *narY*, *nxrB*, *gdhA*, *nrfA*) represents the core conversion nodes of nitrate reduction and nitrite oxidation; Cluster 4 (nitrate reductase auxiliary subunit module) (containing 3 genes, including *narI*, *narV*, *napB*) specifically contains auxiliary subunits of nitrate reductase; Cluster 5 (periplasmic nitrate reduction ammonification module) (containing 2 genes, including *napA*, *nrfH*) involves periplasmic nitrate reduction and ammonification; while Cluster 6 (core denitrification functional module) (containing 5 genes, including *nirK*, *nirS*, *norB*, *norC*, *ureC*) constitutes the core denitrification module. This clear functional modular clustering indicates highly coordinated expression regulation of nitrogen cycle genes, and different treatment conditions are likely to exert differential regulatory effects on the overall nitrogen transformation process through these functional modules.

### 3.5. Association Between Microbial Community, Function, and Environmental Factors

**Figure 6:**
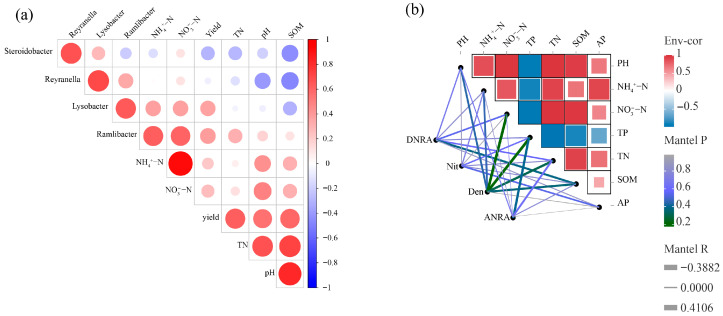
Association Analysis of Microbial Communities, Soil Physicochemical Properties with Crop Yield and Functional Genes. (**a**) Correlations between maize yield and microbial genera as well as soil physicochemical indicators. (**b**) Correlations between nitrogen-cycling functional genes and soil physicochemical indicators.

Based on the correlation data shown in Figure 6a, the indicators demonstrating the most notable positive trends with crop yield are nitrate nitrogen (NO_3_^−^-N, r = 0.354), *Lysobacter* (r = 0.263), and *Ramlibacter* (r = 0.260). These microorganisms form a closely connected synergistic network: *Lysobacter* exhibits a strong positive correlation with *Ramlibacter* (r = 0.793, *p* < 0.01) and a highly synergistic relationship with *Reyranella* (r = 0.837, *p* < 0.01), while *Ramlibacter* is also positively correlated with *Reyranella* (r = 0.585, *p* < 0.05). Collectively, these three genera constitute a core microbial network. This network is primarily driven by key environmental factors: both *Lysobacter* and *Ramlibacter* show significant correlations with ammonium nitrogen (NH_4_^+^-N, r = 0.627, *p* < 0.05; r = 0.630, *p* < 0.05), *Reyranella* is clearly associated with soil organic matter (SOM, r = 0.690, *p* < 0.05), and soil organic matter and pH display an exceptionally strong coordinated variation (r = 0.958, *p* < 0.01). These findings indicate that the microbial members showing positive trends with yield are integral components of a highly synergistic functional microbial community, collectively influenced by key factors within the soil organic matter nitrogen cycle.

Based on the Mantel test analysis between functional genes and environmental factors presented in Figure 6b, denitrification-related genes DNRA and Den exhibit significant environmental responsiveness. The DNRA gene shows a negative correlation with soil organic matter (r = −0.27) and maintains consistent negative correlation trends with various nitrogen forms. Among the five analyzed genes, the Den gene demonstrates the most prominent environmental associations, displaying a positive correlation with total phosphorus (r = 0.41) alongside a negative correlation with nitrate nitrogen (r = −0.39). In contrast, the nitrification gene Nit and other functional genes (ANRA) generally exhibit weaker correlations with environmental parameters. The ANRA gene shows a weak negative correlation with total phosphorus (r = −0.26), while the Nit gene maintains relatively low correlation coefficients across all environmental factors. These correlation patterns indicate that denitrification functional genes respond more strongly to key environmental factors, including carbon, nitrogen, and phosphorus, with the Den gene showing particular sensitivity to environmental variations. This pattern suggests that denitrification processes may play a significant role in responding to environmental element cycling, warranting further investigation into their ecological functions.

## 4. Discussion

### 4.1. Statistically Significant Differences in Soil Physical and Chemical Properties and Crop Yield Under Organic Amendments Treatments

This study systematically evaluated the effects of different organic amendment application methods on key physicochemical properties of maize rhizosphere soil and yield using one-way analysis of variance (ANOVA). The increase in soil pH may be related to the synergistic effects of multi-component materials. Literature indicates that materials such as rice husk ash can release alkaline substances [17], while the decomposition of organic amendments may neutralize soil acidity [18,19]. In the BC treatment, the addition of biochar may have further enhanced the soil’s buffering capacity, resulting in a more pronounced pH elevation.The BC, RS, and DP treatments significantly increased soil ammonium nitrogen (NH_4_^+^-N) and nitrate nitrogen (NO_3_^−^-N) content by 9.10–32.74% and 9.10–29.41%, respectively (*p* < 0.05). The input of organic amendments likely replenished soil available nitrogen through mineralization and nitrification processes [20]. In the BC treatment, biochar, potentially due to its porous structure and other characteristics, may have helped reduce nitrogen loss and created favorable conditions for microbial activity [21,22].

Although the DP treatment significantly increased available nitrogen content by 9.10% (*p* < 0.05), its effect was lower than that of the BC and RS treatments. Under reduced nitrogen conditions, the DP treatment optimized the fertilization timing, but its reliance on chemical nitrogen sources may have led to a different supply pattern in later stages compared to the organic treatments [23,24]. In contrast, the BC and RS treatments achieved continuous nitrogen supply through organic material inputs, with rapeseed cake potentially serving as a fast-release nitrogen source and rice husk possibly acting as a slow-release carrier [25].

This difference in supply patterns may explain why the BC and RS treatments, while maintaining nitrogen availability, significantly increased soil total nitrogen (TN) content by 1.84–3.04% (*p* < 0.05), whereas the DP treatment did not induce a significant change in total nitrogen (*p* > 0.05) [26]. This suggests that under reduced nitrogen conditions, the addition of organic amendments plays an important role in enhancing and sustaining soil nitrogen storage capacity.

The BC and RS treatments significantly increased soil organic matter (SOM) content by 26.20–26.85% (*p* < 0.05). The input of organic materials introduced carbon components with varying stability, which may have influenced the soil carbon pool through mechanisms such as rapid priming effects and long-term inert carbon storage, respectively [27]. Due to its chemical stability, biochar may exhibit greater carbon sequestration potential [28,29].

Collectively, the observed improvements in key soil properties (pH, nitrogen availability and storage, organic matter) under organic amendment treatments demonstrate their significant role in enhancing soil health and building a more resilient soil ecosystem under reduced nitrogen input. Within this context of improved soil health, we observed a positive numerical trend in crop yield following the order DP < RS < BC. This suggests that the combined application of organic amendments represents a promising sustainable soil management strategy, which can effectively enhance soil fertility and, in our study, was associated with a trend of maintained or even slightly improved crop productivity. The primary value of this practice lies in its capacity to improve soil health and nutrient cycling efficiency [30,31].

### 4.2. Organic Substrate-Mediated Differentiation in Microbial Community Abundance

The application of organic amendments significantly altered the microbial community structure in the rhizosphere soil (based on data from this study). The dominant bacterial phyla observed in this study, such as *Acidobacteria* and *Proteobacteria*, whose dominance is consistent with general patterns found in global terrestrial ecosystems [27,32]. Under the reduced nitrogen (DP) treatment, data from this study indicate that the relative abundance of *Bacteroidetes* was higher; according to literature reports, this group is considered to have the potential ability to utilize complex organic nitrogen under low-nitrogen conditions [25,26].

At a finer taxonomic resolution, this study found that the abundance of *Nitrospirae* was significantly lower in the BC and RS treatments. Considering existing literature, *Nitrospirae* are known nitrifying microorganisms, and although organic amendments increased the overall soil pH, local environmental fluctuations may have affected such microorganisms [27]. Data from this study show that the abundance of *Cyanobacteria* showed no significant differences among the treatments. At the genus level, this study found that *Sphingomonas* was significantly enriched in the BC treatment. The combined application of biochar and rapeseed cake may have created a favorable microenvironment for it [28]. Similarly, based on data from this study, *Acidobacterium* maintained a high and stable relative abundance across all treatments, demonstrating broad adaptability to different fertilization regimes.

Furthermore, this study observed a trend of enrichment in genera such as *Bradyrhizobium* and *Lysobacter* under organic amendment treatments. Existing literature points out that these genera have known beneficial functions such as growth promotion and disease suppression [9,33,34,35,36,37,38]. These structural shifts, likely acting in concert with the physicochemical improvements mediated by the amendments, contribute to a more functional and resilient rhizosphere ecosystem [39]. This enhanced soil biological environment supports the role of organic amendments as a sustainable practice for maintaining soil health under reduced nitrogen input [40].

### 4.3. Physicochemical Improvement-Driven Pathway for Organic Amendments Yield Increase: Concurrent with Reduced Microbial Diversity and Formation of Specific Dominant Bacterial Populations

Based on the correlation and Mantel test results from this study, different fertilization treatments may influence nitrogen transformation and crop production by regulating the soil microbial community. Data from this study show that the DP reduced-nitrogen treatment exhibited the highest relative abundance of denitrifying functional genes, which may reflect the potential risks associated with a simple nitrogen reduction strategy. In this treatment, reducing nitrogen input while maintaining the original organic carbon level likely created a high carbon-to-nitrogen ratio environment [41,42]. Existing research suggests that such an environment may promote the proliferation of denitrifying microorganisms [25]. Although, in this study, nitrate nitrogen, *Lysobacter*, and *Ramlibacter*—which are positively correlated with yield—did not dominate in this treatment, the higher abundance of denitrification genes may indicate an increased risk of gaseous nitrogen loss [43].

Results from this study indicate that the BC biochar treatment displayed different regulatory characteristics, with its microbial community being associated with groups such as *Lysobacter*, *Ramlibacter*, and *Reyranella*. These taxa showed correlations with ammonium nitrogen and soil organic matter, suggesting a potential organic matter-pH synergistic effect [44,45]. This study also found that the BC treatment maintained the abundance of genes associated with the denitrification process (Den) at a moderate level. Combined with knowledge from the literature regarding the physical properties of biochar, this may indicate that biochar has a certain regulatory effect on the nutrient environment and microbial processes [46,47]. This moderation of denitrification gene abundance suggests a potential role in balancing nitrogen retention and loss, contributing to a more efficient and environmentally balanced nitrogen cycle within the soil system [48,49].

According to data from this study, the CK conventional fertilization and RS straw treatments shared some similar features, with both exhibiting significantly lower abundance of DNRA genes compared to the DP treatment (Figure 5b). However, neither treatment fostered the dominance of key microbial taxa that are beneficial for soil ecosystem function [50]. The CK treatment provided readily available nitrogen but lacked effective external carbon input, which may have limited the development of relevant functional microorganisms [51]. The straw added in the RS treatment is primarily composed of cellulose and lignin [52,53]. Based on current understanding, its slow decomposition process may not rapidly stimulate the functional activity of the microbial community [54]. The association observed in this study between Reyranella and soil organic matter in the RS treatment might indicate a slowly establishing microbial system [55].

In summary, based on the results of this study, different fertilization treatments drive differentiated microbial responses by altering the carbon-nitrogen balance. The DP treatment suggests that simply reducing nitrogen input could potentially intensify the denitrification process; the CK treatment highlights the limitations of relying solely on chemical fertilizers in cultivating soil microbial functions [56]; the RS treatment represents an improvement pathway dependent on slowly degradable organic materials [57]; and the BC treatment demonstrates a feasible strategy of integrating organic amendments (such as biochar) to synergistically regulate soil physicochemical properties and the microbial community [58]. Collectively, these findings underscore that optimizing the soil environment through measures such as organic amendments—can enhance microbial functionality and nutrient cycling, offering a sustainable approach to maintain soil health and support agricultural productivity under reduced nitrogen input [59].

### 4.4. Limitations

This study also has certain limitations. First, only three field biological replicates were established for each fertilization treatment. While this is a common and practical design in agricultural field experiments, it may limit the statistical power of subsequent multivariate analyses (e.g., PERMANOVA, Mantel tests) and correlation analyses, thereby increasing the risk of Type II errors (i.e., failing to detect true differences or associations) [60]. Additionally, this study did not directly measure key nitrogen cycling process indicators, such as N_2_O emissions or denitrification rates, which may result in an incomplete assessment of the environmental effects of the fertilization treatments [61]. Furthermore, the lack of temporal metagenomic data restricted a deeper analysis of the functional dynamics of microbial communities and their causal mechanisms in relation to the observed phenotypes [62].

In summary, interpretations of trends or relationships that did not reach statistical significance in this study should be made with caution. Future research, where feasible, could consider increasing the number of replicates to enhance statistical power and incorporating more direct process measurements and dynamic metagenomic monitoring. Alternatively, integrating long-term observational data from multiple sites and seasons could further validate and deepen the findings of this study.

## 5. Conclusions

This study elucidates the mechanisms by which organic amendments affect the microbial community and maize yield in Chinese Yellow Earth under reduced nitrogen conditions through field experiments and metagenomic analysis. The results demonstrate that the combined biochar treatment (BC) and the rice husk rapeseed cake treatment (RS) effectively alleviated soil acidification and nutrient deficiency, significantly increasing soil pH, nitrogen components, and organic matter content, thereby enhancing maize yield. Metagenomic analysis further revealed that the BC treatment established a beneficial microbial interaction network centered on *Lysobacter*, *Ramlibacter*, and *Reyranella*. This network was associated with soil ammonium nitrogen and organic matter, forming an organic matter-pH synergistic driving mode. Functional gene analysis indicated that the reduced nitrogen treatment (DP) intensified denitrification, increasing the risk of nitrogen loss. In contrast, the treatment BC maintained denitrification gene abundance at a moderate level through the micro-environmental regulation capacity of biochar, preserving nitrogen transformation functionality while reducing environmental risks. Compared to the conventional fertilization (CK) and RS treatments, which were conducive to nitrogen retention but failed to establish a dominant microbial network positively correlated with yield, the BC treatment demonstrated superior performance. In summary, the combined application of biochar and organic materials under reduced nitrogen conditions, through the synergistic improvement of soil physicochemical properties and the targeted cultivation of beneficial microbial functional networks, represents an effective strategy for achieving green yield increase in the yellow soil region. This provides a theoretical basis for synergistically enhancing productivity and environmental protection through soil microbial management. Based on the findings of this study, a specific practical recommendation is proposed: under the experimental conditions applied here, the combined application of rapeseed cake (2250 kg/ha), rice husk (8010 kg/ha), and biochar (2010 kg/ha)—referred to as the BC treatment—can serve as an effective strategy for balancing maize yield and soil health. In this approach, both rapeseed cake and rice husk are locally sourced agricultural waste materials. The BC treatment achieved a reduction in total nitrogen input of 64.86 kg/ha (approximately 21.39%) compared to conventional fertilization. This strategy not only maintained maize yield with a moderate increase (about 8.97% higher than conventional fertilization) but also significantly improved the pH (by approximately 11.13%) and soil organic matter content (by about 26.20%) in the plough layer of yellow clay soil. It is recommended that further field validation and long-term positioning experiments be conducted in similar local soil types.

## Figures and Tables

**Table 1:** Basic properties of husk, rapeseed cake and biochar.

Organic Amendments	pH	EC (μS/cm)	TC (g/kg)	TN (g/kg)	TP (g/kg)
Rice husk	7.83	136.4	325.88	28.51	3.92
Rapeseed cake	7.33	412.0	412.19	57.14	10.27
biochar	8.07	92.7	635.42	21.45	0.22

**Table 2:** Fertilization Regime and Total Chemical Nutrient Inputs of Different Treatments.

Treatment	Organic Amendments		Base Fertilizer		Seed Fertilizer	Trumpet Fertilizer		Total N	Total P_2_O_5_	Total K_2_O
	Rice Husk	Rapeseed Cake	Biochar	Compound Fertilizer	Ca(H_2_PO_4_)_2_	N 46%				
CK	0	0	0	525	225	210	278	303.23	104.75	78.70
DP	0	0	0	525	225	125	222	238.37	104.75	78.70
RS	8010	2250	0	525	225	125	222	238.37	104.75	78.70
BC	8010	2250	2010	525	225	125	222	238.37	104.75	78.70

Table Note: 1. Application rates for both organic amendments (rice husk, rapeseed cake, biochar) and chemical fertilizers are presented in kg/ha. 2. Total nutrient inputs (Total N, P_2_O_5_, K_2_O) were calculated from chemical fertilizers only; contributions from organic amendments were excluded. 3. Both the RS and BC treatments were implemented on the DP reduced-nitrogen baseline with added organic materials, resulting in identical total chemical nitrogen inputs to the DP treatment. 4. The chemical fertilizer inputs of P_2_O_5_ and K_2_O were consistent across all treatments.

## Data Availability

The raw metagenomic sequencing data generated and analyzed during this study are openly available in the NCBI Sequence Read Archive (SRA) under the BioProject accession number PRJNA1362953 (https://www.ncbi.nlm.nih.gov/bioproject/PRJNA1362953). National Center for Biotechnology Information. n.d. “BioProject PRJNA1362953.” NCBI BioProject. Accessed 18 December 2025. https://www.ncbi.nlm.nih.gov/bioproject/PRJNA1362953.

## Supplementary Materials

The following supporting information can be downloaded at: https://www.mdpi.com/article/10.3390/genes17010009/s1, Table S1: Summary of nitrogen cycle functional gene groups, KEGG gene constituents, and ecological functions. Detailed information on nitrogen cycle gene groups is provided in Supplementary Table S1.
