# Supplementary material for: Enhancing Ecological Functions in Chinese Yellow Earth: Metagenomic Evidence of Microbial and Nitrogen Cycle Reassembly by Organic Amendments
Source: Genes (Basel). 2025 Dec 22;17(1):9. doi: 10.3390/genes17010009 (PMC12840947; doi:10.3390/genes17010009)
Supplement: Supplementary file 1 [file genes-17-00009-s001.zip › genes-4027088-supplementary.pdf]

**Supplementary Materials for "Enhancing Ecological Functions in Chinese Yellow Earth: Metagenomic Evidence of Microbial and Nitrogen Cycle Reassembly by Organic Amendments"**

Table S1. Summary of nitrogen cycle functional gene groups, KEGG gene constituents, and ecological functions.

| **Functional Group（Abbreviation）** | **KEGG Genes Included in the Group** | **Concise Ecological Description** |
|---|---|---|
| Nitrification (Nit) | amoA(K28504); amoB(K10945); amoC(K10946); hao(K10535) | Oxidizes ammonia to nitrite and nitrate. |
| Assimilatory Nitrate Reduction to Ammonium (ANRA) | narB(K00367); nasA, nasC(K00372); nasB(K00360); nirA(K00366); nasD, nasB(K26139); nasE(K26138); nasB(K00361) | Reduces nitrate to ammonium for biomass synthesis. |
| Dissimilatory Nitrate Reduction to Ammonium (DNRA) | narG, narZ, nxrA(K00370); narH, narY, nxrB(K00371); narI, narV(K00374); nirB(K00362); nirD(K00363); napA(K02567); napB(K02568); nrfA(K03385); nrfH(K15876) | Reduces nitrate to ammonium, conserving nitrogen in the soil. |
| Denitrification (Den) | narG, narZ, nxrA(K00370); narH, narY, nxrB(K00371); narI、narV(K00374); napA(K02567); napB(K02568); nirK(K00368); nirS(K15864); norB(K04561); norC(K02305); nosZ(K00376) | Reduces nitrate to gaseous nitrogen ($N_2O$, $N_2$), causing nitrogen loss. |
